# Immunomodulatory Effects of Polysaccharides from *Porphyra haitanensis* in Hydrocortisone-Induced Immunocompromised Mice

**DOI:** 10.3390/foods14061018

**Published:** 2025-03-17

**Authors:** Chunying Du, Chun Wang, Wenwen Zong, Zhaopeng Shen, Peng Wang

**Affiliations:** 1 College of Food Science and Engineering, Ocean University of China, Qingdao 266404, China; 2 Marine Biomedical Research Institute of Qingdao, Ocean University of China, Qingdao 266071, China

**Keywords:** *Porphyra haitanensis* polysaccharides, immunomodulation, hydrocortisone, TLR-4/NF-κB

## Abstract

This study investigated the immunomodulatory effect of polysaccharides from *Porphyra haitanensis* (PHP) using a hydrocortisone-induced immunosuppressive model. Immunocompromised mice were treated with varying doses of PHP and the effects on macroscopic indicators, macrophage function, and both cellular and humoral immune functions were comprehensively assessed. The results showed that PHP significantly increased the body weight and indexes of the spleen and thymus, improved the disorder of blood cell populations, and enhanced macrophage activity. Furthermore, PHP improved T lymphocyte subtypes and differentiation and regulated the CD4^+^/CD8^+^ ratio. PHP also promoted the expression of T-Bet and GATA-3 while maintaining immune homeostasis, alongside promoting cytokine secretion. PHP facilitated the production of antibody-generating cells, serum hemolysin, and antibodies. Western blot results revealed that PHP activates the TLR4/NF-κB pathway. These findings suggested that PHP exerts immunomodulatory effects on both the innate and adaptive immune systems.

## 1. Introduction

The immune system is a sophisticated and essential defense network that protects the body against a wide array of pathogens. Nutritional interventions have become an effective strategy for enhancing both adaptive and innate immunity, ultimately strengthening the body’s disease resistance [1]. Notably, polysaccharides have stood out due to their immunoregulatory properties [2]. *Porphyra* is a genus of red alga comprising various species such as *P. haitanensis*, *P. yezoensis*, and *P. tenera* [3]. It is rich in polysaccharides, which constitute 25%–40% of its dry weight [4]. Recent research has explored the immune-enhancing properties of *Porphyra* polysaccharides, showing their ability to enhance immune responses by stimulating immune cell production and modulating the functions of vital immune cells [5].

Bhatia et al. [6] reported that the oral administration of sulphated polysaccharides from *P. vietnamenis* notably increased the weight of immune organs, boosted the leukocyte and lymphocyte counts, enhanced neutrophil adhesion, and stimulated antibody production, thereby demonstrating their potential immunomodulatory effects. Similarly, the immunoregulatory effects of *P. haitanensis* polysaccharides (PHPS) were verified by their ability to enhance phagocytosis and cytokine secretion in RAW264.7 macrophages [5]. In a BALB/c mice model, PHPS also promoted splenic lymphocyte proliferation and increased the populations of regulatory T cells and dendritic cells. Yoshizawa et al. [7] demonstrated that polysaccharides isolated from *P. yezoensis* stimulated macrophages to produce IL-1 and TNF, while enhancing phagocytic functions both in vivo and in vitro, highlighting their immune-enhancing potential. Moreover, Shi et al. [4] showed that polysaccharides from *P. haitanensis* effectively modulated Th1/Th2 immune response imbalance. Degraded polysaccharides derived from *P. haitanensis* were also found to enhance the immunoregulatory effect in RAW264.7 cells [8].

*P. haitanensis* is predominantly cultivated in the coastal waters of east Asia. Its production constitutes 75% of China’s total laver output and over 50% of global production [9]. The polysaccharides of *P. haitanensis* are sulphated and mainly composed of galactose [10]. Dong et al. [11] employed HPLC to determine the molar ratio of fucose, glucose, and galactose in *P. haitanensis* polysaccharides, which was 1:2.1:76.2. Polysaccharides derived from *Porphyra* have been recognized for their potential immunomodulatory effects. They can enhance immune regulation by stimulating the activities of key immune cells and modulating signaling pathways such as the MAPK and NF-κB [3,12]. However, research on the specific immunomodulatory mechanisms of *P. haitanensis* polysaccharides remains limited.

This study focused on the polysaccharides of *P. haitanensis* (PHP) and aimed to assess their protective effects against hydrocortisone (HC)-induced immunosuppression in a BALB/c mouse model. The immunological activity of PHP was evaluated from multiple perspectives, including non-specific immunity, cellular immunity, and humoral immunity. Additionally, the study explored the immunomodulatory mechanisms by investigating the activation of the TLR4/NF-κB signaling pathway. This study provides a theoretical foundation for the potential application of PHP in functional foods.

## 2. Materials and Methods

### 2.1. Materials

The concanavalin A (ConA) and levamisole hydrochloride (LH) were obtained from Sigma Chemical Co. (St Louis, MO, USA). HC was supplied by Jinyao Pharmaceutical Co., Ltd. (Tianjin, China). Solarbio Technology Ltd. (Beijing, China) supplied the sheep red blood cells (SRBC) and carbon ink. ELISA kits for detecting IL-6, IL-4, TNF-α, IL-2, and IFN-γ were acquired from R&D Co. (Minneapolis, MN, USA). Beckman Coulter, Inc. (Fullerton, CA, USA) supplied the fluorescent microspheres. Flow cytometry antibodies (CD3-APC, CD4-FITC, and CD8-PE) were sourced from BD Biosciences (Franklin Lakes, NJ, USA). Primary antibodies (anti-TLR4, anti-MyD88, anti-NF-κB, anti-p-IκB-α, and anti-GAPDH) and secondary goat anti-rabbit IgG-HRP antibodies were sourced from Cell Signaling Technology (Beverly, MA, USA). Other agents and solvents were of analytical grade.

### 2.2. Preparation and Characterization of PHP

The extraction of *P. haitanensis* polysaccharides (PHP) followed the method reported by Dong et al. [11], with procedural refinements. Briefly, the dried *P. haitanensis* powder was subjected to aqueous extraction (1:25, *w/v*) at 80 °C for 3 h, and clarified via centrifugation (8000 rpm, 10 min). After concentration under reduced pressure, the supernatant was precipitated by adding a three-volume dose of 95% ethanol. Centrifugation was conducted at 4800 rpm for 10 min. The supernatant was eluted through the activated carbon to further purify it; it was then collected and dialyzed for 48 h using a 1 kDa molecular-weight cutoff. Finally, the precipitate was freeze-dried. The yield of PHP was calculated as follows:

(1) $$Yield\left(\%\right)=\frac{Weight\;of\;PHP\;(g)}{Weight\;of\;power\;sample\;(g)}\times 100\%$$

The monosaccharide compositions, protein contents, sulfate contents, and average molecular weight of the PHP were determined.

### 2.3. Animal Experiment

Male BALB/c mice (6–8-weeks old, Vital River Laboratory Animal Center, Beijing, China) were maintained under standard laboratory conditions (23 ± 1 °C, humidity 50 ± 10%, 12 h light–dark cycle) with free access to food and water. After a 7-day acclimatization, the mice were randomly assigned to the following five groups (*n* = 18 per group): Control, HC, LH, PHP-L, and PHP-H. The Control and HC groups received an oral administration of normal saline (0.9%, *w*/*w*), while the LH, PHP-L, and PHP-H groups were given LH 20 mg/kg bw), PHP (90 mg/kg bw), and PHP (270 mg/kg bw), respectively. All treatments were administered once daily for 28 days. From day 23, immunosuppression was induced in the HC, LH, PHP-L, and PHP-H groups through daily intraperitoneal injections of HC (15 mg/kg bw) for 6 days, while the Control group continued to receive saline.

On day 24, six mice from each group were randomly selected to evaluate delayed-type hypersensitivity (DTH), serum hemolysin response test, and antibody-producing cells detection.

On day 29, six mice from the remaining 12 in each group were randomly selected for body weight measurement and blood collection via the angular vein. A portion of the whole blood was collected into EDTA-anticoagulated tubes for hematological analysis and T lymphocyte subset assays. The remaining blood was allowed to clot at room temperature for 30 min and subsequently centrifuged (3000 rpm, 10 min). The separated serum was stored at −80 °C for the determination of cytokines and immunoglobulins. Under sterile conditions, mice were sacrificed; peritoneal macrophages were harvested by lavage with 3 mL of Hank’s solution containing 5% fetal bovine serum. These macrophages were then utilized for the fluorescence microsphere phagocytosis assay. The thymus, spleen, and liver were excised and weighed. The spleen was further processed for subsequent analysis. The remaining six mice in each group were used for the carbon clearance assay.

All protocols were approved by the Animal Experimental Ethics Committee of the College of Food Science and Engineering, Ocean University of China (OUC-AE-2024-004).

### 2.4. Hematological Analysis, Peripheral Blood T Lymphocyte Subset Assay, and Organ Index

The quantities of red blood cells (RBC), hemoglobin (Hb), white blood cells (WBC), platelets (PLT), and lymph (LYMPH) were assessed using an automatic hematology analyzer (MEK-6318K, Nihon-Kohden, Tokyo, Japan).

After treating the blood with a red blood cell lysing solution for 5 min, samples were centrifuged at 1500 rpm for 5 min. Cells were washed three times with PBS and the cell density was adjusted to 1.0 × 10⁶ cells/mL. The cells were then incubated in a blocking buffer containing 20% FBS and CD16/CD32 antibodies (diluted 1:100) at 4 °C for 30 min. Following the blocking step, 100 μL of the cell suspension was stained with 20 μL of the antibodies (CD8^+^/CD4^+^/CD19^+^) for 30 min in the dark [13]. After two washes with PBS, flow cytometric analysis was performed by using a FACS Aria III cytometer (BD Bioscience, San Jose, CA, USA).

The thymus and spleen were weighed and the organ index was calculated using the following formula:

(2) $$Organin\;dex\;(mg/g)=\frac{Organ\;weight\left(mg\right)}{Body\;weight\left(g\right)}$$

### 2.5. Detection of Phagocytic Activity

The phagocytic activity of peritoneal macrophages was evaluated using fluorescent microspheres, following a method modified from that of Lin et al. [14]. The 1 mL suspension of macrophage (4–6 × 10^7^ cells/mL) was incubated with fluorescent microspheres (1 × 10^7^ microspheres/well) under conditions of 37 °C and 5% CO_2_ for 2 h. When the incubation was complete, cells were washed twice with PBS and analyzed. The phagocytic rate was calculated as follows:

(3) $$Phagocytic\;rate\left(\%\right)=\frac{Number\;of\;macrophages\;containing\;microspheres}{Total\;number\;of\;macrophages}\times 100\%$$

### 2.6. Spleen T Lymphocyte Proliferation Assay

The MTT assay was used to evaluate the proliferation of splenic T lymphocytes [15]. Splenocytes were isolated by grinding the spleen in sterile saline and passed it through a 200-mesh filter. The cell suspension was treated with sterile distilled water for 5 s and then centrifuged at 1000 rpm for 5 min, followed by three washes with sterile saline. The splenocytes were resuspended in RPMI-1640 complete medium, which was supplemented with 10% fetal bovine serum. A volume of 100 μL of the splenocyte suspension (5 × 10^6^ cells per/mL) was added to each well of the 96-well plates. ConA, at a concentration of 5 μg/mL, was added. An unstimulated blank control without ConA was also used. The culture plate was placed in a 5% CO_2_ incubator at 37 °C for 72 h. Then, 20 μL of the MTT solution (5 mg/mL) was added and incubated for an additional 4 h. The supernatant was carefully aspirated, and 150 μL of DMSO was added. The cell proliferation was evaluated by measuring the absorbance at 570 nm.

### 2.7. Delayed-Type Hypersensitivity (DTH), Serum Hemolysin Response Test and Antibody-Producing Cells Detection

On day 24, mice were intraperitoneally injected with 0.2 mL SRBC (2%, *v*/*v*). On day 28, the thickness of the right hind footpad was measured and the same antigen was administered via subcutaneous injection. On day 29, the footpad thickness was measured again; the change in thickness was used to evaluate delayed-type hypersensitivity (DTH) [16].

Blood was collected and allowed to clot; serum was separated by centrifugation (4000 rpm for 10 min). The serum samples were then diluted 250-fold with SA buffer, which was prepared as follows: 0.252 g NaHCO_3_, 0.46 g C_4_H_4_N_2_O_3_, 0.2 g CaCl_2_, 0.1 g MgCl_2_, 0.3 g C_8_H_11_ N_2_NaO_3_, and 8.38 g NaCl, made up to 1 L with distilled water. A total of 100 μL of the diluted serum, 50 μL of SRBCs (10%, *v*/*v*), and 100 μL of fresh guinea pig serum (diluted 1:8 with SA buffer) were mixed and incubated at 37 °C for 30 min. The mixture was then cooled on ice and centrifuged at 1500 rpm for 10 min. An aliquot of 50 μL of the supernatant was transferred to a 96-well plate, followed by the addition of 150 μL of Dush’s solution. A blank control was prepared by substituting the serum sample with SA buffer. Meanwhile, the 12.5 μL of SRBC was mixed with 187.5 μL of Dush’s solution. The absorbance at 540 nm was recorded, and the half value of the hemolysis (HC50) was calculated with the following formula:

(4) $$HC50=\frac{OD1-OD3}{OD2-OD3}\times serum\;dilution\;factor$$

where OD1, OD2, and OD3 represent the absorbance values of the sample, SRBC, and blank control, respectively.

The antibody-producing cells were detected using the quantitative hemolysis spectrophotometry (QHS) method. Under sterile conditions, the spleen was removed, and the splenocyte suspension was prepared according to Section 2.6. The splenocytes were resuspended at a concentration of 2 × 10^7^ cells/mL. An 0.5 mL aliquot of the splenocyte suspension was mixed with 0.4 mL of SRBC and 0.5 mL of fresh guinea pig serum was added. A control group was prepared by replacing splenocytes with saline. The mixture was kept for 60 min at 37 °C, followed by centrifugation (3000 rpm, 5 min). The supernatant was collected; absorbance was measured at 413 nm.

### 2.8. Carbon Clearance Assay

The phagocytic activity of macrophages was evaluated by carbon clearance assay, following the method described by Li et al. [17]. On day 29, all groups were administered a tail vein injection of a carbon ink suspension at a dose of 0.1 mL/10 g body weight, with the suspension diluted fourfold with saline. Blood samples were drawn from the retro-orbital plexus at 2 min and 10 min post injection. The samples were combined with 2 mL of 0.1% Na_2_CO_3_ and the related absorbance was recorded at 600 nm. Finally, mice were sacrificed and the livers and spleens were all separated and weighed. The phagocytic index was calculated as follows:

(5) $$Phagocytic\;index=\sqrt[{3}]{\frac{lgOD2min-lgOD10min}{10min-2min}}\times \frac{body\;weight\;(g)}{liver\;weight\left(g\right)+spleen\;weight\;(g)}$$

### 2.9. Serum Cytokines and Immunoglobulins Determination

The levels of IFN-γ, IL-2, TNF-α, IL-4, IL-5, IgA, IgM, and IgG in the serum were analyzed using ELISA kits, following the manufacturers’ protocols.

### 2.10. Quantification of Acid Phosphatase (ACP) and Lactate Dehydrogenase (LDH) Activities

The spleen samples collected in Section 2.3 were homogenized in cold PBS at a concentration of 10% (*w*/*v*). The homogenate was subsequently centrifuged (2500 rpm, 10 min) at 4 °C and the supernatant was then collected. The activities of ACP and LDH were determined using the Acid Phosphatase Assay kit (Beyotime, Shanghai, China) and the Lactate Dehydrogenase Activity Assay kit (Sigma-Aldrich, St. Louis, MO, USA), respectively, according to the related manufacturers’ protocols.

### 2.11. Quantitative Real-Time PCR (qRT-PCR) Analysis

Total RNA was isolated from the spleen using UNIQ-10 column total RNA purification kit (Sangon Biotech Co., Ltd., Shanghai, China).The qRT-PCR was conducted following the method described by Wang et al. [18]. The expression of β-actin was used as an internal reference; the primers utilized are listed in Table 1.

### 2.12. Western Blot Analysis

The spleen tissues were homogenized on ice using lysis buffer and subsequently centrifuged to collect the supernatant. The protein concentrations were measured using a BCA protein assay kit. The quantified proteins were separated using 10% SDS–PAGE and then transferred onto a polyvinylidene difluoride (PVDF) membrane. The membrane was blocked with 5% non-fat powdered milk for 3 h at room temperature and then incubated overnight at 4 °C with primary antibodies. Following a wash with TBST buffer, the membrane was incubated with secondary antibodies conjugated to horseradish peroxidase for 3 h. Immunoreactive bands were detected using an enhanced chemiluminescence kit (Applygen, Beijing, China) and Chemi-Doc XRS system. Band quantification was performed with ImageJ 1.54f software (National Institutes of Health), with GAPDH as the internal control.

### 2.13. Statistical Analysis

The data are presented as mean ± standard deviation (SD) and were analyzed using SPSS 18.0 software (SPSS Inc., Chicago, IL, USA). A one-way analysis of variance (ANOVA) with Dunnett’s test was employed to evaluate the statistical significance. Significance was defined as *p* < 0.05 or *p* < 0.01.

## 3. Results and Discussion

### 3.1. Characterization of PHP

The PHP was isolated from *P. haitanensis* and its chemical compositions and properties were characterized. The polysaccharide extraction yield was 18.9%, with a sulfate content of 1.86 mg/mL. The PHP contained 0.071 g/L protein and its molecular weight was approximately 8.3 × 10^5^ Da. The monosaccharide composition revealed a molar ratio of 1.00:84.74:3.10 for glucose, galactose, and fucose, respectively, indicating that galactose is the predominant component of PHP.

Sulfated polysaccharides are characterized by their ability to modulate immune function. The sulfate groups in these polysaccharides are believed to interact with immune receptors, thereby influencing immune responses [19]. Liu et al. [5] reported that sulfated polysaccharides derived from *P. haitanensis*, which contain 14.67% sulfate, effectively exhibited immunoregulatory properties. Additionally, Yuan et al. [20] demonstrated that polysaccharides with a galactose content of 45 mol% could bind to TLR2 receptors in RAW 264.7 cells, promoting nitric oxide production and cytokine secretion. These processes are crucial for enhancing immune responses against infections. The unique combination of sulfate groups and galactose in PHP suggested that this polysaccharide may possess immunomodulatory properties.

### 3.2. Effect of PHP on Body Weight, Immune Organ Indexes, and Hematological Parameters

**Figure 1:**
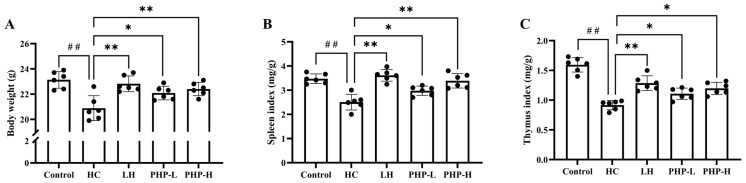
Effect of PHP on body weight (**A**); spleen index (**B**); and thymus index (**C**) in HC-treated mice. Data are presented as mean ± SD (*n* = 6). Each dot represents a biological sample. One-way analysis of variance (ANOVA) with Dunnett’s test was used to assess statistical significance among different groups. Statistical significance was set at *p* < 0.05. ^##^ *p* < 0.01 vs. Control group. * *p* < 0.05 and ** *p* < 0.01 vs. HC group.

As shown in Figure 1 and Table 2, the HC group exhibited weight loss (*p* < 0.01), spleen and thymus indexes changes (*p* < 0.01), and blood cell populations disturbance (*p* < 0.05) compared to the Control group. These alterations were consistent with the established immune suppression model, where HC administration led to impairments in immune function [18,21]. Therefore, this study successfully constructed an HC-induced immunosuppressive mouse model.

In the PHP groups, particularly the PHP-H group, body weight showed significant improvement compared to that of the HC group (*p* < 0.01). The spleen index in the PHP-H group also exhibited notable recovery (*p* < 0.01), returning to near normal levels. Similarly, the thymus index was enhanced in the PHP-treated groups (*p* < 0.05). The administration of LH and PHP led to varying degrees of recovery, with significant increases in RBC counts (*p* < 0.01). LH notably improved WBC and LYMPH levels (*p* < 0.05), while PHP demonstrated even greater enhancements (*p* < 0.01). In the PHP-H group, RBC, WBC, Hb, and PLT levels increased by 13.38%, 65.04%, 11.85%, and 19.83%, respectively (*p* < 0.01), effectively restoring these parameters to normal levels. The overall health and immune function of mice can be assessed through body weight, immune organ indexes, and hematological parameters [6]. PHP administration effectively alleviated HC-induced changes in macroscopic indicators in a dose-dependent manner. This provides evidence of its immune-modulatory capabilities. Polysaccharides derived from other species, such as *P. vietnamenis* [6] and *P. yezoensis* [3], have also demonstrated similar enhancements.

### 3.3. Effect of PHP on Macrophage Function

**Figure 2:**
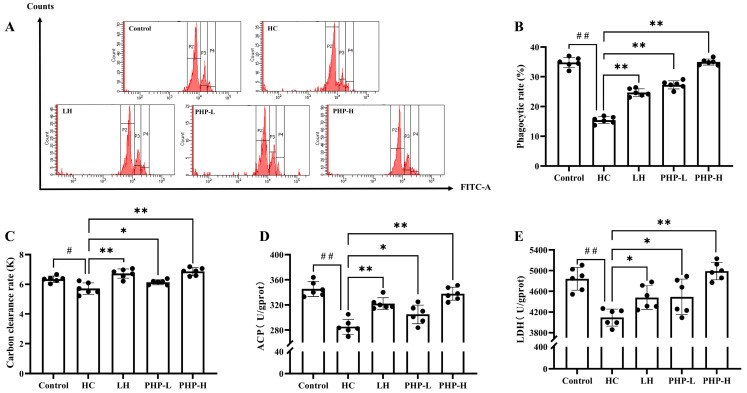
Effect of PHP on macrophage function in HC-treated mice: (**A**) flow cytometry histogram plots of the fluorescent microspheres engulfed by spleen macrophages; (**B**) phagocytic rate of macrophages; (**C**) carbon clearance rate; (**D**) the activities of ACP; and (**E**) the activities of LDH. Data are presented as mean ± SD (*n* = 6). Each dot represents the mean of technical triplicates for each biological sample. One-way analysis of variance (ANOVA) with Dunnett’s test was used to assess statistical significance among different groups. Statistical significance was set at *p* < 0.05. ^#^ *p* < 0.05 and ^##^ *p* < 0.01 vs. Control group. * *p* < 0.05 and ** *p* < 0.01 vs. HC group.

As depicted in Figure 2A,B, the phagocytic activity of macrophages in the PHP-treated groups significantly increased (*p* < 0.01) in a dose-dependent manner compared to that of the HC group. In the PHP-L and PHP-H groups, the phagocytic rate rose by 10.60% and 41.66%, respectively, compared to that of the LH group. The carbon clearance rate is closely linked to macrophage phagocytic function. PHP-H treatment significantly reversed the HC-induced negative effect (*p* < 0.01), surpassing the level observed in the Control group (Figure 2C). Marker enzymes, such as ACP and LDH, are often used to assess the level of activation in macrophages [22]. As shown in Figure 2D,E, treatment with LH and PHP effectively prevented the decrease in LDH and ACP activities induced by HC. Notably, both LH and high-dose PHP significantly increased ACP and LDH levels compared to that of the HC group, with ACP rising by 12.92% (*p* < 0.01) and 18.39% (*p* < 0.01), and LDH by 9.45% (*p* < 0.05) and 21.95% (*p* < 0.01), respectively.

Macrophages are pivotal in both specific and non-specific immunity, playing essential roles in pathogen clearance, immune regulation, and the secretion of cytokines [23]. Their functional status can be assessed through indicators such as phagocytic activity, carbon clearance, and intracellular enzyme levels. This study revealed that PHP enhanced macrophage activation and function. Polysaccharides from *P. haitanensis* have been shown to enhance the phagocytic capacity of RAW 264.7 macrophages [8]. The *P. vietnamenis* polysaccharides increased the carbon clearance rate in immunosuppressed mice [6]. Moreover, Wu et al. [22] reported a significant elevation of LDH and ACP levels in response to polysaccharide treatment, suggesting a critical role in supporting macrophage function. These studies emphasized the immune-enhancing effects of natural polysaccharides, further supporting the potential of PHP as a promising immunomodulatory agent.

### 3.4. Effect of PHP on Cellular Immune Response

**Figure 3:**
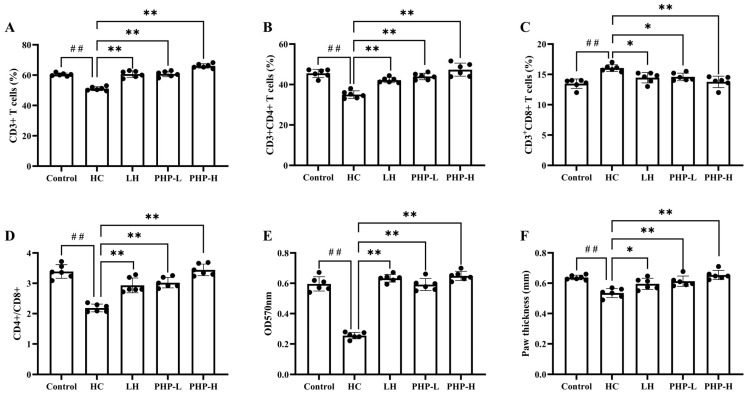
Effect of PHP on cellular immune response in HC-treated mice: (**A**) peripheral blood CD3^+^ T cell subset; (**B**) peripheral blood CD3^+^CD4^+^ T cell subset; (**C**) peripheral blood CD3^+^CD8^+^ T cell subset; (**D**) CD4^+^/CD8^+^ ratio; (**E**) spleen T lymphocyte proliferation; (**F**) delayed-type hypersensitivity. Data are presented as mean ± SD (*n* = 6). Each dot represents the mean of technical triplicates for each biological sample. One-way analysis of variance (ANOVA) with Dunnett’s test was used to assess statistical significance among different groups. Statistical significance was set at *p* < 0.05. ^##^ *p* < 0.01 vs. Control group. * *p* < 0.05 and ** *p* < 0.01 vs. HC group.

T lymphocytes are central to cellular immunity; their proliferation and subsets are closely related to immune function [24,25]. CD4^+^ T cells mainly regulate antibody production and aid in the activation of B cells, enhancing immune responses against pathogens, while CD8^+^ T cells focus on eliminating infected or abnormal cells [26]. They are both differentiated from CD3^+^ T cells. As shown in Figure 3A–C, high-dose PHP treatment elevated the levels of CD3^+^ T cells and the CD4^+^ subpopulation and reduced the proportion of the CD8^+^ subpopulation (*p* < 0.01). Additionally, the CD4^+^/CD8^+^ ratio in the PHP-L and PHP-H groups significantly increased, by 38.14% (*p* < 0.01) and 57.52% (*p* < 0.01), respectively, compared to that of the HC group (Figure 3D). The CD4^+^/CD8^+^ ratio is a key indicator of immune status, with a decline often associated with immunosuppression and severe disease [27]. Restoring this balance has been recognized as a crucial therapeutic strategy in managing immune disorders, as demonstrated by Jie et al. [28]. In this study, the shift in the CD4^+^/CD8^+^ ratio observed with PHP treatment suggests its potential to enhance T cell-mediated immune responses, which are crucial for maintaining immune homeostasis.

ConA serves as a mitogen that stimulates T lymphocyte transformation and proliferation [29]. The proliferation of spleen cells in response to ConA stimulation serves as an important indicator of cellular immune function [30]. In this study, spleen T lymphocytes were isolated and treated with ConA, as illustrated in Figure 3E. HC treatment markedly inhibited spleen T lymphocyte proliferation, reducing it by 42.79% compared to that of the Control group (*p* < 0.01). Both LH and PHP effectively counteracted this suppression (*p* < 0.01) and outperformed the Control group. In addition, PHP exhibited a dose-dependent enhancement, with the PHP-H group exhibiting superior effects to those of the LH group. Furthermore, DTH is a comprehensive cellular immune response driven by antigen-specific T cells, particularly CD4^+^ and CD8^+^ T cells, and can be evaluated via the footpad swelling test [31]. As shown in Figure 3F, treatment with LH and PHP led to a significant increase in footpad swelling (*p* < 0.05 and *p* < 0.01, respectively), with high-dose PHP producing the most pronounced effect. This result underscored the ability of PHP to stimulate antigen-specific T cell responses, further supporting its role in strengthening cellular immunity.

Macrophages serve as antigen-presenting cells. Upon encountering pathogens or other immune triggers, macrophages engulf and process these antigens, presenting fragments of them on their surface in the context of major histocompatibility complex (MHC) molecules [13]. This antigen presentation is essential for the activation of naive T cells. The increased phagocytic activity and carbon clearance rate observed in PHP-treated groups enhanced the ability of macrophages to efficiently process and present antigens. This could facilitate T cell activation, leading to the proliferation and differentiation of T cells, thereby initiating and amplifying adaptive immune responses. Promoting the regeneration of CD4^+^ T cells may be an option for immune reconstruction [32]. Additionally, macrophages express co-stimulatory molecules, like CD80 and CD86, which are necessary for the full activation of T cells [33]. Investigating how PHP influences macrophage-mediated antigen presentation could provide deeper insights into its immunomodulatory mechanisms.

### 3.5. Effect of PHP on mRNA Levels of T-Bet and GATA3 and Secretion of Serum Cytokines

**Figure 4:**
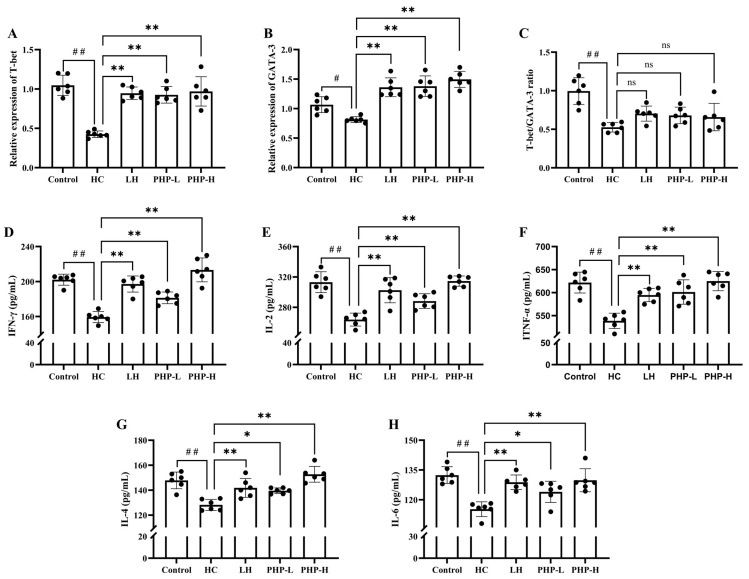
Effect of PHP on mRNA levels of T-Bet and GATA3 in spleen and serum cytokines in HC-treated mice: (**A**) relative expression level of T-Bet; (**B**) relative expression level of GATA-3; (**C**) T-Bet/GATA-3 ratio; (**D**) level of IFN-γ; (**E**) level of IL-2; (**F**) level of TNF-α; (**G**) level of IL-4; and (**H**) level of IL-6. Data are presented as mean ± SD (*n* = 6). Each dot represents the mean of technical triplicates for each biological sample. One-way analysis of variance (ANOVA) with Dunnett’s test was used to assess statistical significance among different groups. Statistical significance was set at *p* < 0.05. ^#^ *p* < 0.05 and ^##^ *p* < 0.01 vs. Control group. * *p* < 0.05 and ** *p* < 0.01 vs. HC group.

T-Bet and GATA3 are key transcription factors driving CD4^+^ T cell differentiation into Th1 and Th2 subsets, respectively. An imbalance between Th1 and Th2 cells is often linked to inflammatory and autoimmune disorders [34]. Previous studies have shown the ability of polysaccharides to regulate T-Bet and GATA-3 expression, thereby restoring the Th1/Th2 balance [35]. As illustrated in Figure 4A–C, HC treatment led to a significant reduction in the expression levels of T-Bet (*p* < 0.01) and GATA3 (*p* < 0.05), accompanied by a marked decrease in the Th1/Th2 ratio (*p* < 0.01). Treatment with either LH or PHP effectively restored this suppression, significantly increasing T-Bet (*p* < 0.01) and GATA3 (*p* < 0.01) expression levels. However, the T-Bet/GATA3 ratio showed no significant difference compared to the HC group.

The cytokine profiles provided further insights into the modulatory effects of PHP. Cytokines are synthesized and secreted by activated immune cells. They mediate the communication between immune cells, regulating both innate and adaptive immunity. Specifically, cytokines, such as IFN-γ, IL-2, and TNF-α, are associated with Th1 cells, while IL-4 and IL-6 are key cytokines for Th2 cells [36]. From the results in Figure 4D–H, treatment with HC significantly inhibited the secretion of these cytokines (*p* < 0.01). After the administration of LH or PHP, the levels of IFN-γ, IL-2, TNF-α, and IL-4 were significantly reversed (*p* < 0.01), approaching the levels observed in the normal control group. The observed reversal of cytokine secretion aligns with the restored transcriptional activity of T-Bet and GATA3.

Beyond antigen presentation, macrophages interact with T cells through cytokine secretion, further influencing their activation and differentiation. Activated CD4^+^ T cells, in turn, secrete cytokines like IFN-γ, which enhance macrophage phagocytic activity, forming a feedback loop crucial for effective immune response regulation [37]. The impact of PHP on mRNA expression and cytokine production revealed its capacity to broadly activate the immune system rather than selectively modulate CD4^+^ T cell subset polarization. Although the restoration of the T-Bet/GATA3 ratio was incomplete, this indicated that PHP enhanced overall T cell function without completely re-establishing the balance between Th1 and Th2 cells. By modulating the secretion of Th1- and Th2-associated cytokines, PHP demonstrated a potential role in maintaining Th1/Th2 homeostasis.

### 3.6. Effect of PHP on the Humoral Immune Response

**Figure 5:**
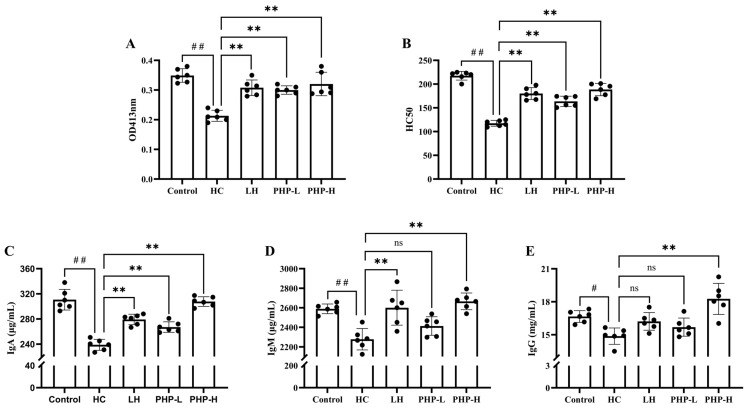
Effect of PHP on the humoral immune response in HC-treated mice: (**A**) antibody-producing cells detection; (**B**) serum hemolysin response; (**C**) level of IgA in serum; (**D**) level of IgM in serum; and (**E**) level of IgG in serum. Data are presented as mean ± SD (*n* = 6). Each dot represents the mean of technical triplicates for each biological sample. One-way analysis of variance (ANOVA) with Dunnett’s test was used to assess statistical significance among different groups. Statistical significance was set at *p* < 0.05. ^#^ *p* < 0.05 and ^##^ *p* < 0.01 vs. Control group. ** *p* < 0.01 vs. HC group.

The present study examined the effect of PHP on humoral immunity in HC-immunosuppressed mice, with significant findings observed in several key immune markers. As shown in Figure 5A, compared to the HC group, PHP treatment significantly promoted the secretion of antibody-forming cells, with an increase of 1.41-fold (*p* < 0.01) and 1.50-fold (*p* < 0.01) in the PHP-L and PHP-H groups, respectively. This suggests that PHP treatment effectively enhanced B cell activation and differentiation. Consistent with the QHS results, LH and PHP treatments significantly elevated serum hemolysin levels (Figure 5B). Moreover, treatment with 270 mg/kg bw PHP significantly restored serum IgA, IgG, and IgM levels (*p* < 0.01), outperforming the positive effects observed in the LH group (Figure 5C–E). This suggests that PHP treatment effectively enhanced B cell activation and differentiation.

The humoral immune response, which is crucial for defense against extracellular pathogens, is largely mediated by antibodies produced by B cells. This process is tightly regulated by the activation and differentiation of B cells and the production of various immunoglobulins (Ig), including IgA, IgM, and IgG. Yu et al. [38] showed that extracts of *Cyclina sinensis* can dose-dependently increase CD4^+^ T lymphocytes, restore the CD4^+^/CD8^+^ ratio, restore DTH, and elevate serum levels of IgA, IgG, IgM, and hemolysin. As reported by Huang [39], the restoration of the Th1/Th2 balance can accelerate the formation of serum hemolysin. The findings from the present study, along with previous reports, highlight the potential of PHP as an effective immunomodulatory agent. Further investigations are warranted to explore the fine structure of PHP and its structure–activity relationship.

### 3.7. Effect of PHP on the TLR-4/NF-κB Pathway

Recent research has begun to elucidate the specific mechanisms by which *Porphyra* polysaccharides modulate immune responses. For instance, two *Porphyra* polysaccharides have demonstrated immunomodulatory activity, primarily through NF-κB-dependent pathways that promote immune cell maturation and differentiation [3]. Gong et al. [12] reported that purified polysaccharide (PHPD-IV-4) extracted from *P. haitanensis*, significantly increased the phosphorylation of ERK1/2, P38, and JNK in a dose-dependent manner. This finding indicated that PHPD-IV-4 enhanced macrophage immunomodulatory function by upregulating MAPK pathway activity. Furthermore, *P. haitanensis* polysaccharides have been shown to activate the JNK and JAK2 signaling pathways in RAW 264.7 cells, leading to elevated iNOS mRNA expression and increased NO production [5].

**Figure 6:**
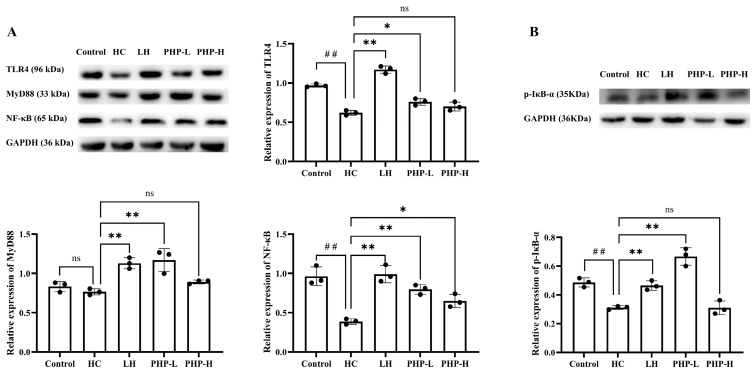
Effect of PHP on the protein expression of key factors in the TLR4/NF-κB pathway from the spleen: (**A**) the protein expression levels of TLR4, MyD88, and NF-κB; and (**B**) the protein expression level of p-IκB-α. Data are presented as mean ± SD (*n* = 3). Each dot represents a biological sample. One-way analysis of variance (ANOVA) with Dunnett’s test was used to assess statistical significance among different groups. Statistical significance was set at *p* < 0.05. ^##^ *p* < 0.01 vs. Control group. * *p* < 0.05 and ** *p* < 0.01 vs. HC group.

The NF-κB signaling pathway plays a crucial role in mediating immune and inflammatory processes, impacting both innate and adaptive immunity [40]. MyD88 is a critical adaptor protein in this cascade, facilitating the activation of NF-κB as its main downstream effector [41]. Once phosphorylated, IκB-α undergoes degradation, promoting the release and nuclear translocation of NF-κB, which subsequently activates the expression of immune-related genes [42]. As shown in Figure 6, compared to the Control group, HC treatment led to the downregulation of TLR4, MyD88, p-IκB-α, and NF-κB expression. HC suppressed immune reactions by inhibiting the activation of the TLR-4/NF-κB pathway. Conversely, treatment with LH (*p* < 0.01) and PHP-L (*p* < 0.01) effectively restored and upregulated the expression of these key signaling molecules, suggesting the reactivation of this critical pathway. A previous study demonstrated that the activation of the TLR4/NF-κB pathway enhances macrophage function, promoting innate immune responses [43]. Moreover, NF-κB activation has been shown to facilitate T cell differentiation and increase the CD4^+^/CD8^+^ ratio, strengthening adaptive immunity [18]. A study has shown that the TLR4 signaling pathway mediates the proliferation and differentiation of immune cells and the secretion of cytokines [44].Consistent with these findings, PHP demonstrated an immunomodulatory effect in immunosuppressed mice, closely associated with the activation of the TLR4/NF-κB signaling pathway.

## 4. Conclusions

Polysaccharides, especially those derived from *P. haitanensis*, have shown promising immunomodulatory effects. In this study, the immunomodulatory effects and underlying mechanism of PHP were investigated. The results demonstrated that PHP significantly enhanced immune function by activating the TLR-4/NF-κB signaling pathway, leading to macrophage activation and increased cytokine production. This activation of macrophages subsequently promoted T cell proliferation and differentiation, while also boosting B cell activation and antibody production. Together, these effects enhanced both cellular and humoral immunity. These findings suggest that PHP is a candidate for enhancing immune responses.

## Figures and Tables

**Table 1:** Primers used for mRNA expression analysis.

Genes	Forward (5′→3′)	Reverse (5′→3′)
*β-Actin*	TGTCCACCTTCCAGCAGATGT	AGCTCAGTAACAGTCCGCCTAGA
*T-Bet*	CCTCAATACCCGCCCAAGATG	CATGGGCAGAGTTCGCATGG
*GATA-3*	CAGGCAGGGAGTGTGTGAAC	GCATTGCAAAGGTAGTGCCC

**Table 2:** Effect of PHP on hematological parameters in HC-treated mice.

Groups	RBC (10^13^/L)	WBC (10^9^/L)	Hb (g/L)	PLT (10^11^/L)	LYMPH (10^9^/L)
Control	1.01 ± 0.10	3.64 ± 0.19	157.07 ± 2.32	8.81 ± 0.31	2.56 ± 0.24
HC	0.92 ± 0.08 ^##^	2.33 ± 0.36 ^##^	140.62 ± 3.55 ^#^	7.55 ± 0.11 ^##^	1.05 ± 0.23 ^##^
LH	1.05 ± 0.17 **	2.98 ± 0.12 *	151.50 ± 4.24 *	8.42 ± 0.09 *	1.42 ± 0.31 *
PHP-L	1.02 ± 0.14 **	3.09 ± 0.17 **	151.00 ± 3.16 *	8.19 ± 0.18	1.73 ± 0.15 **
PHP-H	1.08 ± 0.23 **	3.73 ± 0.26 **	158.83 ± 5.21 **	9.17 ± 0.24 **	2.13 ± 0.21 **

Data are presented as mean ± SD (*n* = 6), with technical triplicates performed for each biological sample. One-way analysis of variance (ANOVA) with Dunnett’s test was used to assess statistical significance among different groups. Statistical significance was set at *p* < 0.05. ^#^ *p* < 0.05 and ^##^ *p* < 0.01 vs. Control group. * *p* < 0.05 and ** *p* < 0.01 vs. HC group.

## Data Availability

The original contributions presented in the study are included in the article. Further inquiries can be directed to the corresponding authors.
